# NATpare: a pipeline for high-throughput prediction and functional analysis of nat-siRNAs

**DOI:** 10.1093/nar/gkaa448

**Published:** 2020-05-28

**Authors:** Joshua Thody, Leighton Folkes, Vincent Moulton

**Affiliations:** School of Computing Sciences, University of East Anglia, Norwich NR4 7TJ, UK; School of Biological Sciences, University of East Anglia, Norwich NR4 7TJ, UK; School of Computing Sciences, University of East Anglia, Norwich NR4 7TJ, UK

## Abstract

Natural antisense transcript-derived small interfering RNAs (nat-siRNAs) are a class of functional small RNA (sRNA) that have been found in both plant and animals kingdoms. In plants, these sRNAs have been shown to suppress the translation of messenger RNAs (mRNAs) by directing the RNA-induced silencing complex (RISC) to their sequence-specific mRNA target(s). Current computational tools for classification of nat-siRNAs are limited in number and can be computationally infeasible to use. In addition, current methods do not provide any indication of the function of the predicted nat-siRNAs. Here, we present a new software pipeline, called NATpare, for prediction and functional analysis of nat-siRNAs using sRNA and degradome sequencing data. Based on our benchmarking in multiple plant species, NATpare substantially reduces the time required to perform prediction with minimal resource requirements allowing for comprehensive analysis of nat-siRNAs in larger and more complex organisms for the first time. We then exemplify the use of NATpare by identifying tissue and stress specific nat-siRNAs in multiple *Arabidopsis thaliana* datasets.

## INTRODUCTION

**Figure 1.**
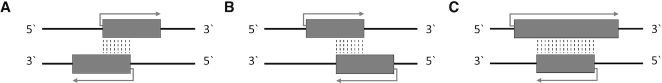
The three types of *cis*-NAT orientation that can form dsRNA: 5′ overlap (head-to-head) (**A**), 3′ overlap (tail-to-tail) (**B**) and the complete enclosure of one transcript by the other (full overlap) (**C**). Transcript sequences are always transcribed in the 5′ direction and are represented by arrows. Regions of complementarity between the two sequences are represented by dashed lines.

Natural antisense transcripts (NATs) are endogenous RNA transcripts that share sequence complementary to other RNA transcript sequences (1). They have been identified in multiple eukaryotes, including humans, mice, yeast, rice and Arabidopsis (2). NATs include both protein coding and, mostly, non-protein coding transcripts (3) and can be classified into either *cis*-NATs or *trans*-NATs based on their genomic origin. *cis*-NATs are transcribed from the same genomic location but on opposite strands, resulting in sections of perfectly complementary double stranded RNA (dsRNA) forming between the two transcript sequences. Conversely, *trans*-NATs originate from different genomic locations and can form imperfect dsRNA (2). There are three types of *cis*-NAT orientation that can form dsRNA: 5′ overlap (head-to-head), 3′ overlap (tail-to-tail) and the complete enclosure of one transcript by the other (full overlap) (3), shown in Figure 1. Although current understanding is limited, research has suggested a variety of regulatory roles for NATs, such as RNA interference (RNAi), alternative splicing, genomic imprinting, and X-chromosome inactivation (2,4,5).

Small RNAs (sRNAs) are short, non-coding RNAs that are vital components of gene regulation acting through endogenous RNA silencing pathways (6). They regulate many important and diverse biological pathways such as growth and development, disease resistance and stress response (7,8). To do this, they suppress the translation of messenger RNAs (mRNAs) by directing the RNA-induced silencing complex (RISC) to its sequence-specific mRNA target(s). In plants, there are many well categorized classes of sRNA, such as microRNA (miRNA), small interfering RNA (siRNA), heterochromatic small interfering RNA (het-siRNA) and trans-acting short interfering RNA (ta-siRNA), differentiated by both biogenesis and mode of action (9). One mechanism of sRNA-mediated mRNA regulation is through mRNA cleavage and the resulting mRNA fragments can be captured on a genome-wide scale using degradome sequencing techniques, such as Parallel analysis of RNA ends (10) or nanoPARE (11). This data can then be used to support sRNA target prediction by aligning the captured cleavage fragments back to the reference transcript sequences which are then used to identify possible causal sRNAs (12–15). Furthermore, this data can also be used to capture cleavage products generated through Dicer-mediated processing of sRNA precursors, as previously demonstrated with miRNA biogenesis (13,16,17).

Over the last few years, much research attention has been focused on the biogenesis and function of natural antisense transcript-derived small interfering RNAs (nat-siRNAs) (5,18–21). The founding example was identified in *Arabidopsis thaliana*, where a pair of *cis*-NATs, SRO5 and P5CDH, were shown to be involved in the response to salt tolerance through the RNAi pathway (5). During salt stress, SR05 is expressed and can form a complementary overlapping region with the constitutively expressed P5CDH, which is then processed by a biogenesis pathway dependent on Dicer-like 2 (DCL2), RNA-dependent RNA polymerase 6 (RDR6), Suppressor of Gene Silencing 3 (SGS3) and DNA-directed RNA polymerase IV subunit 1 (NRPD1) to produce a 24nt nat-siRNA. This nat-siRNA then directs the cleavage of P5CDH, which is subsequently used as a template by RDR6 to produce dsRNA that is then processed by DCL1 to produce 21nt secondary nat-siRNAs (5).

Recently, NATs were identified in public sequencing data from 69 plant species and a database called PlantNATsDB (22) was constructed. This database includes information regarding sRNAs originating from overlapping and non-overlapping regions of NAT transcript pairs. In 2012, *Zhang et al*. performed a genome-wide analysis of plant nat-siRNAs in both *Oryza sativa* and *A. thaliana*, which revealed insights into their distribution, biogenesis and function (21). In this study, >17 000 unique siRNAs corresponding to cis-NATs from biotic and abiotic stress challenged *A. thaliana* and 56 000 from abiotic stress treated *O. sativa*. These siRNAs were enriched in the overlapping region of NAT pairs and displayed either site-specific or distributed patterns.

Current tools available for the prediction of nat-siRNAs are limited in both number and functionality. NATpipe (23), a collection of Perl scripts developed for the prediction of NATs and nat-siRNAs, is the only computational pipeline for this type of analyses, however it suffers from limitations in its runtime and also requires a large number of third-party dependencies that must be installed and configured by the user. This requires computational expertise that some users may not have. Additionally, NATpipe is developed to exclusively discover phased-distributed nat-siRNAs, however based on a previous study (21), nat-siRNAs production can also follow site-specific patterns and thus would be missed by NATpipe. Moreover, the results reported by NATpipe do not give any indications into the possible function of any predicted nat-siRNAs. Finally, based on our prediction performance benchmarking, limitations with the implementation of the NATpipe algorithm causes some known *cis*-NAT pairs and their corresponding *cis*-nat-siRNAs to be discarded.

In this paper, we introduce NATpare, a tool for the prediction and functional analysis of nat-siRNAs. NATpare takes sRNA, transcriptome and optionally, degradome, data as input and enables the identification of both *cis*- and *trans*-nat-siRNAs. It is scalable with the increasing size of modern sequencing datasets and enables comprehensive analysis of nat-siRNAs in more complex transcriptomes for the first time within a reasonable time frame. In addition, if corresponding degradome data is available, NATpare provides the reported nat-siRNAs to PAREsnip2 (24) for prediction of potential mRNA targets based on evidence within the degradome.

## MATERIALS AND METHODS

**Figure 2.**
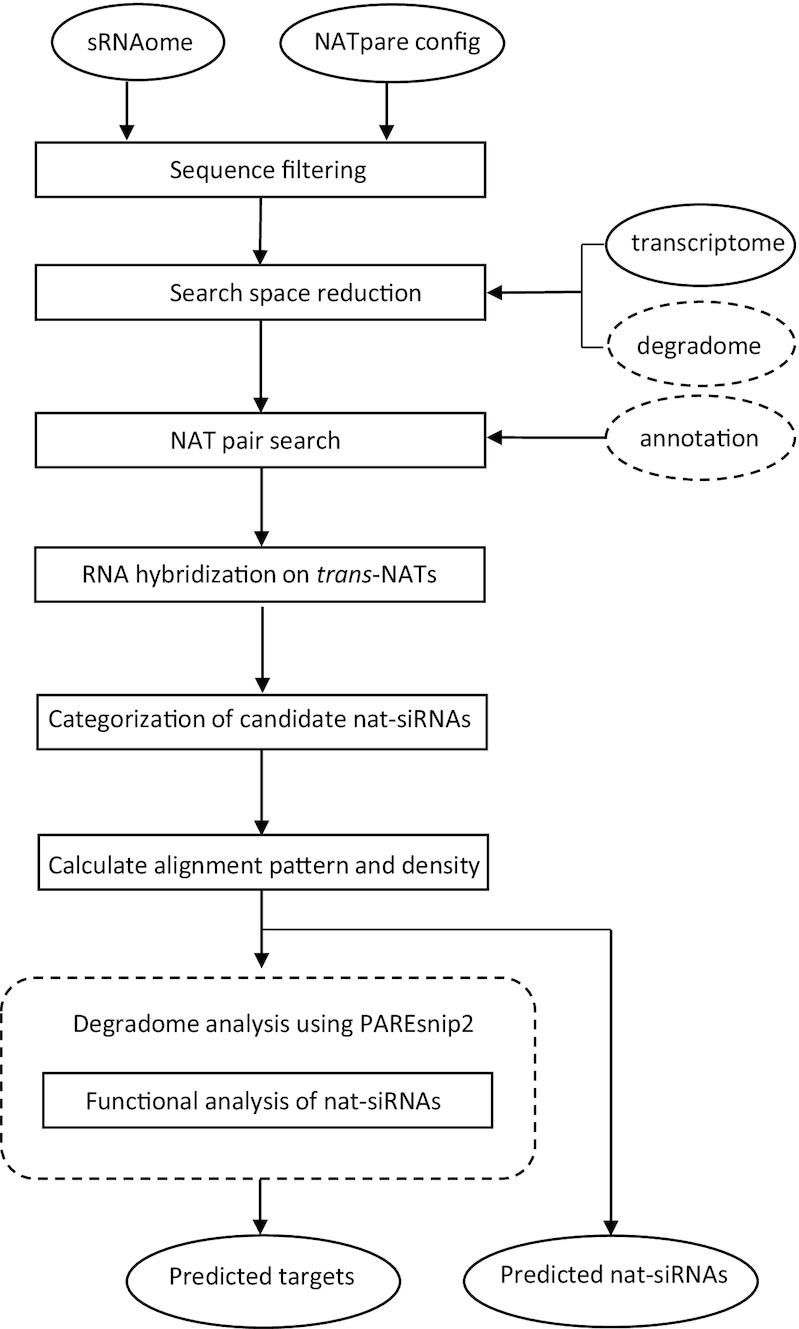
A visual overview of the NATpare pipeline. Input and output data are represented by ovals and processes are represented by rectangles. Data input or processing steps surrounded by dashed lines are optional and dependent on the provided input data. NATpare takes as input HTS data (sRNA and degradome) along with a reference transcriptome and outputs a list of predicted nat-siRNA. Additional annotation information, in the form of a GFF3 file, can be used to annotate the predicted NATs (*cis* or *trans)* by incorporating genomic origin.

The NATpare algorithm is split into four main stages, with the final stage being optional and dependent on the input data. The first is the pre-processing of input sequencing data and the approaches taken to reduce the possible search space. The second stage is the identification of potential NAT pairs. In the third stage, potential nat-siRNAs are identified and additional quantitative information is extracted and reported. Finally, and if degradome data is provided, the candidate nat-siRNAs are subject to functional analysis using PAREsnip2 (24) to search for potential mRNA targets. A visual overview of the steps involved in performing analysis on the input data is shown in Figure 2. We now explain each stage of the algorithm in more detail.

### Data input and tool configuration

To perform an analysis using NATpare for a specific organism, the user must input the following data:

- A reference file (transcriptome) in either FASTA or Generic Feature Format version 3 (GFF3) with corresponding genome;
- A genome file (optional unless using GFF3 as reference);
- A set of sRNAs in redundant FASTA format;
- A degradome library in redundant FASTA format (optional).

A reference file and at least one sRNA library are required to perform analysis. If the user chooses to use a GFF3 file as a reference then a corresponding genome must also be provided. When extracting the gene sequences from the genome using information from the provided annotation (GFF3), the tool will include all splice variants of a given transcript that are detailed within the annotation. The input sRNA library must be in redundant FASTA format with the adaptors trimmed. Tools available to processing FASTQ files, such as adaptor trimming and other quality checking, can be found in the UEA sRNA Workbench (25), where NATpare is also included.

**Figure 3.**
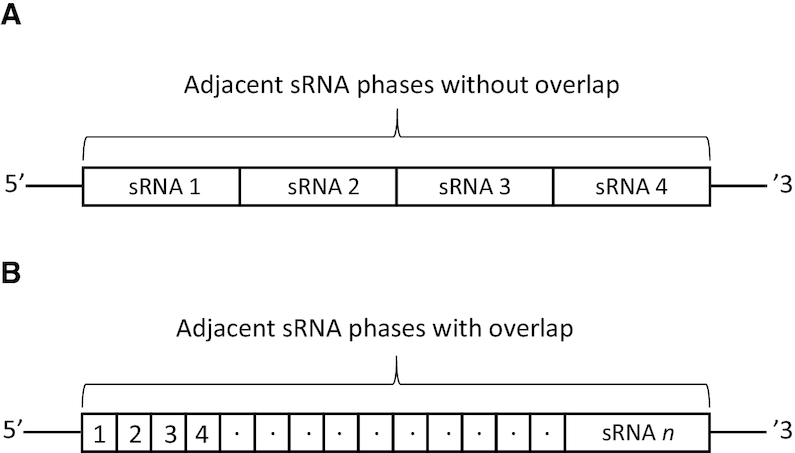
The two types of adjacent sRNA alignment phases considered by NATpare. Adjacent sRNA phases without overlap (**A**) are when the first position at the 5′ end of an aligned sRNA is adjacent to the last position at the 3′ end of another aligned sRNA. Adjacent sRNA phases with overlap (**B**) are where sRNA sequences align contiguously to a given transcript.

When performing analysis, the user has the option to configure a number of parameters to meet their requirements, which are shown in Table 1. The most notable parameters are the number of expected sRNA phases, which is defined as the number of expected adjacent sRNAs, with or without overlap, that align to a given transcript for it to be reported, as shown in Figure 3, and the minimum overlap length between two NATs (i.e. the minimum overlap length considered possible to produce sRNAs).

**Table 1.** The configurable parameters for NATpare. The values used during analysis can be changed by modifying the input configuration file or by using the command line when running the tool.

Parameter	Default value	Description
Minimum overlap length	100	Minimum length of the annealed region between NATs
Minimum sRNA phases	1	Minimum number of sRNA alignment phases (shown in Figure 3)
Minimum sRNA length	19	Minimum input sRNA length
Maximum sRNA length	24	Maximum input sRNA length
Minimum sRNA abundance	1	Minimum input sRNA abundance
Minimum tag length	19	Minimum length of degradome reads
Maximum tag length	21	Maximum length of degradome reads
*Cis* only	true	Only search for NATs with perfectly complementary or from the same genomic location
Coverage ratio	80%	The percentage of overlap required between the BLAST and RNAplex alignments
Largest bubble region	10%	Largest non-complementary region in a *trans*-NAT alignment cannot be longer than 10% of the total alignment
Low complexity filter	true	Discard input sequences based on their complexity
Genome alignment	true	If a genome is provided, discard any sRNAs that do not align

### Sequence filtering

Several optional filtering techniques can be applied to the input data to remove low quality reads, sequencing errors or sample contamination. First, any sequence containing ambiguous bases are discarded as they cannot be accurately aligned. Second, a low complexity sequence filter is applied based on the sequence composition (24). Specifically, this works by discarding any sequences that contain more than 75%, 37.5% and 25% of a single, di- or tri-nucleotide composition, respectively. Finally, if a genome is provided, sRNA sequences can be aligned using PatMan (26), with any sequences that do not align being discarded.

### Search space reduction

A core component of the NATpare algorithm is the pre-processing of the input data to reduce the possible search space and thus reduce the required runtime of a given analysis. In the first step, the sRNA and optional degradome libraries are aligned to the provided transcript sequences in the positive direction with no mismatches allowed. For this, we use the Binary Search Alignment algorithm implemented in UEA sRNA Workbench (24,25). Next, we extract sub-sequences based on the following criteria:

- Adjacent aligned sRNA sequences, either at the 5′ end or 3′ end, that meet the minimum number of expected phases (configurable parameter).
- If provided, degradome tags where the first position aligns adjacent to the 3′ position of an aligned sRNA, which results in a ∼40nt sequence.

The use of degradome data is to find DCL cleavage evidence and to determine those sRNA that may be site-specific, e.g. there is a preferential DCL cleavage site, based on the types of distribution patterns found in a previous study (21).

Once the longer sub-sequences, that meet either of the above criteria, have been extracted, we take their reverse complement and perform exact match sequence alignment to all other transcripts using PatMan (26). This process gives us potential overlapping regions, that give rise to sRNAs, between two transcripts and are then subject to more a comprehensive investigation.

### NAT pair search

After the generation of the candidate NATs from the search space reduction technique, they are subject to an alignment search using BLAST (27). If the alignment length is greater than or equal to the expected minimum, the NAT pair is then identified as either *cis* or *trans*. If a GFF3 file is provided as input this will be determined by the genomic origin of the two transcripts, otherwise it will be determined based on previously described criteria (23). Specifically, if the overlapping region is perfectly complementary, it will be considered as a *cis*-NAT, otherwise it will be considered as a *trans*-NAT albeit the lack of genomic location information. In the case of *trans*-NATs, the reported alignment is further analysed using RNAplex (28) to verify the annealing potential of the BLAST-predicted alignment at the secondary structure level. The results from RNAplex must meet the following criteria for the NAT pair to be considered for further analysis:

1. The reported annealing region should overlap with the BLAST reported complementary region by at least 80% (configurable parameter)
2. Any unpaired region within the annealing region should be no longer than 10% (configurable parameter) of the total length of the overlapping region

Unlike NATpipe, we only do the hybridization analysis if the reported BLAST alignment or genomic location information suggests that the NAT pairs work in *trans*. In addition, to compensate for the long processing time of RNAplex, if the length of either transcript of the NAT pair is greater than 5000nt, we omit the hybridisation step and instead just proceed with the reported BLAST alignment.

Once all of the candidate NATs have been processed, those passing all the required criteria are categorized into the following groups:

- High-coverage (HC): the complementary region is longer than 50% of the length of either transcript
- 100nt: the complementary region is 100nt or longer in length
- Low-coverage (LC): the complementary region is less than 100nt in length

### Categorization of candidate nat-siRNAs

Once the overlapping regions between NATs have been determined, the pipeline extracts the sRNA sequences that aligned to these positions. Rather than just providing the user with a set of aligned sRNAs, we developed a system to categorise each sRNA based on the current understanding of the sRNA biogenesis model. For this system, we also include degradome data (if provided) as this provides a snapshot of the mRNA degradation profile, which can include Dicer-mediated cleavage products. In addition, by looking at the degradation profile, it can also give us an indication as to what mRNAs are currently being expressed, as the mRNA must be expressed in order to be degraded, and thus improve our nat-siRNA prediction model.

For each biogenesis group, we define the mature sRNA as the one originating from the transcript currently being investigated. For example, given the NAT pair consisting of transcripts A and B, when investigating sRNA alignments to transcript A, those sRNAs aligning to B will be considered the star sequences, and vice-versa when investigating transcript B, those aligning to A will be considered the star sequence.

Below is an overview of the biogenesis groups:

- Group 1: sRNA and sRNA* sequence present with 2 nt 5′ overhang and both sequences supported by the degradome data
- Group 2: sRNA and sRNA* sequence present with 2 nt 5′ overhang and only the mature sequence supported by the degradome data
- Group 3: sRNA and sRNA* sequence present with 2 nt 5′ overhang
- Group 4: sRNA present with degradome evidence but no sRNA*
- Group 5: Only the sRNA aligning to the overlapping region

### NAT alignment distribution and sRNA alignment densities

To determine the distribution pattern of aligned sRNAs for a given NAT pair, we implemented a method described previously (21). Specifically, starting from the first aligned sRNA closest to the 5′ end of a transcript, sRNAs were clustered if their first nucleotide is within a 10nt long segment of the starting sRNA, with any cluster containing more than 5 reads being retained for further analysis. For each NAT, we record the number of clusters and the percentage of the unique reads in these clusters relative to the whole transcript. Alignments are considered to be site-specific if a transcript contains 10 or less clusters and the percentage of unique reads within these clusters is 50% or greater than that over the whole transcript, otherwise it is categorized to have a distributed pattern.

For each NAT pair, we also report the sRNA alignment density for the overlapping region and also for the whole transcript. To do this, we implement the same methods as described previously (21). Briefly, for each NAT, we counted the number of unique sRNAs, denoted as N_o_, mapping to the overlapping region and the total number, denoted as N_g_, mapping to both transcripts. We then measured the length of the overlapping region, denoted as L_o_, and the sum of the length of both transcripts, denoted as L_g_. Finally, the ratios N_o_/L_o_ and N_g_/L_g_ were reported as the sRNA alignment densities for the overlapping region and for the overall transcript sequences within the NAT pair, respectively.

### Functional analysis of candidate nat-siRNAs

It has been shown that *cis*-nat-siRNAs can direct the cleavage of their mRNA targets (5). Therefore, to provide further indication of the function of the reported nat-siRNAs and if degradome data is provided as input, we incorporate the predicted nat-siRNAs into PAREsnip2 (24). For the target prediction, we allow the user to configure their own parameters or alternatively use the default configurations provided by PAREsnip2. Additionally, if the user has a version of R installed and is correctly configured as an environment variable, the pipeline can automatically produce t-plots to provide a visual representation of the reported interactions.

### Implementation and output

The algorithm has been implemented using the Java programming language and a user-friendly, cross-platform software package has been incorporated into the UEA sRNA Workbench (25). Analysis using NATpare can be performed through the command-line interface as a standalone application or alternatively be incorporated into larger and more complex bioinformatics pipelines or workflows.

The results of NATpare are provided in comma-separated value (CSV) format, allowing them to be viewed in any CSV file viewer.

### Sequence datasets

To enable a comprehensive evaluation of the NATpare tool, we performed computational benchmarking on five plant species with varying transcriptome sizes (Supplementary Table S1), namely *A. thaliana*, *S. lycopersicum*, *O. sativa*, *G. max* and *T. aestivum*. The transcriptome used for all species in the computational performance benchmarking were extracted from genome and GFF files, obtained from plant Ensembl (29) release 43. 100 000 sRNA sequences were used in the computational benchmarking for each species and were simulated from the overlapping region of two randomly selected cis-NATs, based on the genomic information provided within the genome annotation. All generated sequences were 21nt in length and were randomly selected to be extracted from either transcript within the NAT pair. This benchmarking was performed on a desktop computer running Ubuntu 18.04 equipped with a 3.40 GHz Intel Core i7-6800K six core CPU and 128GB RAM.

For the prediction comparison between NATpipe and those reported by a previous study, we used the publicly available *G. max* sRNA dataset, which we will refer to as D1 hereafter, obtained from a previous study (30) (GEO accession GSE33380), and the reference transcriptome obtained from the Phytozome database (31) release 12. For all *A. thaliana* analysis, besides from the computational benchmarking, we used the TAIR10 reference transcriptome obtained from The Arabidopsis Information Resource (TAIR) (32). The control and stress treated *A. thaliana* sRNA sequences that were used for the seedling salt stress analysis were obtained from a previous study (33) (GEO accession GSE66599) and will be referred to as dataset D2 hereafter. The *A. thaliana* flower, root, seedling and leaf libraries, with corresponding degradome data, were generated from plants grown at 21C and obtained from NCBI BioProject PRJNA407271 (34) and will be referred to as dataset D3 hereafter.

## RESULTS

### Benchmarking and comparison with NATpipe

To measure the computational performance of the newly developed NATpare algorithm, i.e. the time and memory required to perform an analysis, we carried out computational benchmarking and compared our results to those of the other publicly available method.

For this benchmarking, we used the simulated set of sRNA sequences and the reference transcriptome, produced using the GFF file obtained from Ensembl (29), as described in the methods, for each species. The reason that we used simulated data is that it allows us to generate nat-siRNAs that we know should be captured by the tools and thus allows for the fairest possible comparison. As NATpipe can only predict nat-siRNA originating from *cis*-NATs, we adjusted the NATpare parameters to also have this restriction. We recorded the time taken for each tool to perform analysis on the simulated data and the results of these analyses are shown in Table 2. If the tool did not complete the analysis within 10 days, we recorded it as did not finish (DNF). The results show that the newly developed algorithm substantially outperforms NATpipe on the simulated datasets in terms of computation time. For the *A. thaliana* dataset, the only dataset that NATpipe was able to complete within the 10 day cut-off limit, the newly developed method was able to complete the analysis with over 200× speed up. For all tested datasets, the memory requirement varied between 4GB and 8GB depending on the number of transcript sequence within the reference annotation. The timing results suggest that the time taken is dependent on the number of transcripts and transcript pairs that contain overlapping and complementary regions, for which the exact number is difficult to determine, particularly when you consider *trans*-NATs, as this information is not possible to obtain, even with a complete genome annotation, without thorough computational analysis. However, the results of the computational performance benchmarking demonstrate NATpipe's speed limitations and the need for additional pipelines or software tools for the prediction of nat-siRNAs.

**Table 2.** Computation performance comparison between NATpipe and the newly developed NATpare pipeline when evaluated on 5 simulated datasets. If the tool did not finish within 10 days it was recorded as did not finish (DNF).

Species	Annotation version	# Transcripts	NATpipe time	NATpare time
*S. lycopersicum*	SL3.0	33925	DNF	4 min 52 s
*O. sativa*	IRGSP-1.0	42378	DNF	5 min 38 s
*A. thaliana*	TAIR10	48359	1 day 18 h 34 min	11 min 15 s
*G. max*	G. max v2.1	88412	DNF	1 h 5 min
*T. aestivum*	IWGSC	133744	DNF	13 h 2 min

Next, we wanted to evaluate the predictions reported by the tools on real sequencing data. However, unlike other classes of sRNA, such as miRNAs, there is no extensive set of true positives. Nevertheless, a number of previous studies have manually predicted NATs and nat-siRNAs in both model and non-model plants, for example, *A. thaliana* (21), *G. max* (30) and *Z. mays* (35). As NATpipe is currently the only publicly available tool for the prediction of nat-siRNAs, we performed an analysis on a publicly available *G. max* dataset and investigated the overlap in the number of nat-siRNAs reported by computational methods, NATpipe and NATpare, and those found previously during manual analysis (30). For this analysis, we used the *G. max* cDNA reference transcriptome, obtained from Phytozome and the D1 sRNA dataset, as described in the methods. In addition, to compensate for the long processing time required by NATpipe and the fact that it is only able to predict *cis*-nat-siRNAs, we restricted the input transcript sequences only to those with perfectly complementary overlapping regions, as reported by a BLAST search using those transcripts previously found to produce nat-siRNAs (30) as input.

The results from this analysis, with the top 10 NAT pairs based on number of generated nat-siRNAs, presented in Table 3 and the rest in Supplementary Table S2, show that NATpare is able to capture a larger number of the previously reported nat-siRNAs in *G. max* compared to NATpipe. To investigate the overlap in results between the two tools, we compared the results and found that all of the NATpipe reported nat-siRNA were a subset of those reported by NATpare. In addition, further investigation into the NAT pairs missed by NATpipe showed that the RNAplex hybridization step of the algorithm did not always correspond to the alignment reported by BLAST, thus no results were reported, which supports our decision to perform RNA hybridization exclusively on *trans*-NATs. Interestingly, we observed differences between the numbers of reported nat-siRNAs from the previous study (30) and the prediction tools and consider this likely to be a result of minor discrepancies between the different filtering and prediction methods applied to the input sRNAs.

**Table 3.** Top 10 reported *G. max cis*-NATs with the highest number of unique reported nat-siRNAs by Zheng *et al.* (30) and the prediction results from NATpare and NATpipe.

Gene A	Gene B	Overlap length	Zheng *et al.* reported sRNAs	NATpipe	NATpare
Glyma13g11940.1	Glyma13g11970.1	542	1864	0	1802
Glyma13g11820.1	Glyma13g11830.1	428	1285	0	1406
Glyma13g11940.1	Glyma13g11950.1	147	724	0	576
Glyma13g11940.1	Glyma13g11960.1	118	509	0	487
Glyma11g30060.1	Glyma11g30070.1	392	244	209	237
Glyma13g21780.1	Glyma13g21790.1	355	28	0	28
Glyma15g06490.1	Glyma15g06500.1	156	26	0	26
Glyma17g23860.1	Glyma17g23870.1	174	18	11	11
Glyma03g22390.1	Glyma03g22400.1	276	17	16	17
Glyma15g37470.1	Glyma15g37480.1	764	15	0	15

### Comparing the expression of nat-siRNAs in *A. thaliana* control and salt stress treated samples

The current understanding of NATs and nat-siRNAs is that they are expressed during certain stress conditions, development stages or disease response (5,21,36). To illustrate the use of NATpare and to validate the results reported by the tool, we performed analysis on a publicly available dataset, D2, obtained from *A. thaliana* seedling under salt stress, a type of abiotic stress in which the plants response has been previously shown to involve nat-siRNAs (5). Before performing analysis using NATpare and to increase confidence within the predictions, we discarded any sRNAs that were not conserved between at least 2 out of 3 biological replicates. Next, we further filtered the data to remove any known miRNAs or isomiRs by aligning the sRNAs to all known plant miRNAs, obtained from miRBase (release 22) (37), allowing up to 2 mismatches. In addition, we removed any sRNAs that may have originated from tRNA or rRNA sequences using the filtering methods implemented within the UEA sRNA Workbench (25). The results for this analysis, including the breakdown of the NAT and nat-siRNA prediction categories, can be found in Supplementary Table S3. After performing analysis on the filtered data using NATpare, we then investigated the overlap between the control and treatment samples and the results show that there exists a clear separation in the reported nat-siRNAs between treatment and control, with just 281 overlapping sequences within the intersection, yet 877 and 581 being specific to control and treatment, respectively. As the biogenesis of nat-siRNAs require both transcripts to be expressed simultaneously within the same cell, the separation and differences in the number of nat-siRNAs that are reported between control and treatment may be due to transcriptional changes in response to the stress.

To investigate these results further, we performed differential expression analysis with iDEP (38), using the default parameters, which reported 31 differentially expressed (DE) nat-siRNAs using a false discovery rate of 0.1. These comprised of 29 upregulated nat-siRNAs in the treatment datasets, presented in Table 4, and two up-regulated nat-siRNA in the control datasets. For each of the upregulated nat-siRNAs identified in the treatment datasets, we examined the current annotation model (TAIR10) and found that 10 of the 29 sequences originated from a NAT pair where one of the transcripts is currently annotated as a potential natural antisense gene. Majority of the other upregulated nat-siRNAs in the treatment datasets originated from transcripts annotated as either unknown protein or other RNA. Further analysis of all NAT pairs giving rise to these nat-siRNAs, besides for AT5G01600.1 and AT5G01595.1, showed that the sRNA alignment density within the overlapping region was greater than that of the whole transcript, suggesting that sRNAs are more likely to originate from overlapping regions of these NATs.

**Table 4.** The upregulated nat-siRNAs, as reported by iDEP, in the *A. thaliana* seedling salt-stress dataset. Ten of the 29 sequences originated from NAT pairs where one of the transcripts is annotated as a potential natural antisense gene. The transcript that gives rise to the largest number of nat-siRNAs is currently annotated as ‘unknown RNA’ and the corresponding NAT has an unknown function. Adjusted *P*-values were obtained using a false discovery rate of 0.1 and were expressed to three significant digits. Any extreme *P*-values (i.e. *P* < 0.001) were reported as *P* < 0.001.

Sequence	Originating gene	Originating gene annotation	Corresponding NAT	Corresponding NAT annotation	log_2_fc	Adjusted *P*-value
CAAAAACTGCTGAATCGTCGAGG	AT3G41761.1	other RNA	AT3G41762.1	unknown protein	7.759025974	*P* < 0.001
CCGGCGACTTTTCCGGCGATCGG					7.728742081	*P* < 0.001
CAAAAACTGCTGAATCGTCGAGGA					6.425292703	*P* < 0.001
AAAAACTGCTGAATCGTCGAGG					6.214796612	*P* < 0.001
AAAAACTGCTGAATCGTCGAGGA					6.133011199	*P* < 0.001
CCGGCGACTTTTCCGGCGATCGGT					5.961226293	*P* < 0.001
CGGCGACTTTTCCGGCGATCGG					5.539642282	*P =* 0.002
CCGGCCGCCGGGATTTTCGCCGG					5.283876137	*P =* 0.007
AAAAACTGCTGAATCGTCGA					4.989198969	*P =* 0.035
GGCGACTTTTCCGGCGATCGG					4.908960699	*P =* 0.062
CCGGCCGCCGGGATTTTCGCCG					4.22132342	*P =* 0.061
GGCGACTTTTCCGGCGATCG					4.117655813	*P =* 0.081
AACTGCTGAATCGTCGAGG					3.689930846	*P =* 0.035
TCCGGCGACTTTTCCGGCGATCGG					3.580577335	*P =* 0.001
AAAACTGCTGAATCGTCGAGG					3.080787251	*P =* 0.044
CCGGCCGCCGGGATTTTCGCC					2.703061225	*P =* 0.027
AAACTGCTGAATCGTCGAGGA					2.517183992	*P =* 0.054
CAAAAACTGCTGAATCGTCGAG					2.435469953	*P =* 0.002
TAAGAGAGAACAAGGATGGTT	AT1G05560.1	UDP-glucosyltransferase 75B1	AT1G05562.1	Potential natural antisense gene	4.458736007	*P =* 0.035
GACAAGTAGAAAAAAAATGGCG					3.790780596	*P =* 0.026
AGTAGAAAAAAAATGGCGCCA					3.258296457	*P =* 0.007
CAAGTAGAAAAAAAATGGCGCC					3.16407171	*P* < 0.001
AAGTAGAAAAAAAATGGCGCC					2.086703913	*P =* 0.024
CAAGTAGAAAAAAAATGGCGC					1.98426758	*P =* 0.027
TGAGAATTTTCGGTTTGGTTT	AT1G05562.1	Potential natural antisense gene	AT1G05560.1	UDP-glucosyltransferase 75B1	5.178982904	*P =* 0.015
TTGTTTGTGTTGGAAGGTGTG					4.804480168	*P =* 0.098
AGACAGATTAGGTAACTCGAA					2.199439073	*P =* 0.035
GCGGCGGAGAAGTATGTGGATA	AT3G59068.1	Potential natural antisense gene	AT3G59070.1	Cytochrome b561/ferric reductase transmembrane with DOMON related domain	4.908960699	*P =* 0.062
GCCACTACTCCCTCACGGCTCTGC	AT5G01600.1	ferretin 1	AT5G01595.1	other RNA	6.220625993	*P* < 0.001

### Investigation into the function of *cis-* and *trans*-nat-siRNAs in different Arabidopsis tissues

In a previous study by Yuan *et al.* (39), manual analyses of 40 publicly available *A. thaliana* sRNA datasets obtained from flower, leaf and seedling tissues identified 5385 nat-siRNAs that could be mapped to the overlapping region of a single *cis-* or *trans*-NAT pair and were conserved between at least three of the 40 datasets. Of these, 1548 were found to be conserved between each tissue whereas 945 and 142 were specific to seedling and flower, respectively. Analyses into the function of nat-siRNA has shown that they can act as post-transcriptional gene regulators, like miRNAs, by directing the RISC to sequence-specific mRNA targets, usually in *cis* (5,40). Degradome data provides experimental support that increases confidence with sRNA target prediction (41) and the NATpare pipeline includes PAREsnip2 for target prediction and functional analysis of reported nat-siRNA candidates. To illustrate the usefulness of combining prediction with functional analysis, we performed analysis using NATpare on the D3 dataset, which consists of two synonymous *A. thaliana* sRNA and degradome biological replicates obtained from each flower, leaf, root and seedling.

For this analysis, and similar to the analysis performed in a previous study (39), we configured NATpare to report both *cis-* and *trans*-nat-siRNAs. Similar to our previous analysis, we removed any sRNAs that were not conserved between both replicates and also removed predictions that aligned to any known miRNA, rRNA or tRNA sequences using the UEA sRNA Workbench (25). After performing analysis on the filtered sRNAs (Supplementary Table S4), we further processed the results to remove any predicted nat-siRNAs that were reported to originate from multiple transcripts. In total, there were 2962, 1505, 2701, 3562 nat-siRNAs candidates reported in flower, leaf, root and seedling, respectively. We then investigated the overlap between the nat-siRNAs reported from each tissue and found that 613 nat-siRNAs (9.6% of all reported sequences) were conserved between each of the tissues. The tissue with the largest number of uniquely reported nat-siRNAs was seedling, with 1438 (22.6% of all reported sequences), and the tissue with the fewest uniquely reported sequences was leaf with just 272 (4.3% of total reads). These results are consistent with those reported by Yuan *et al.* (39), where it was also found that seedling tissue produces the largest number and leaf tissue produces the smallest number of unique nat-siRNAs. A Venn diagram, created by InteractiVenn (42), showing the overlap between all tissues within the D3 dataset can be found in Supplementary Figure S1. Further analysis into the nat-siRNA candidates found that 96.5%, 98.5%, 98.1% and 97.6%, of nat-siRNAs identified in flower, leaf, root and seedling, respectively, were uniquely reported in this study, when compared to those previously reported (39).

To identify the possible function of the captured nat-siRNAs, we performed target prediction with PAREsnip2, using default targeting criteria but without additional filtering (Supplementary Table S5), on the dataset D3 degradome libraries. The sRNA input for degradome analysis on each tissue were the captured nat-siRNAs that passed all filtering methods described above. The results of each analysis can be found within Supplementary Table S6. A *t*-plot showing an exemplary interaction that was reported in seedling is shown in Supplementary Figure S2. The time taken to perform target prediction on each dataset was ∼5 min with a peak memory usage of 4GB. After performing analysis on each dataset, we extracted the reported targets that were conserved between each of the replicates. This resulted in 6 targets from 4 nat-siRNAs captured in flower, 29 targets from 8 nat-siRNAs captured in leaf, 63 targets from 29 nat-siRNAs captured in root and 35 targets from 9 nat-siRNAs captured in seedling. To exemplify the use of degradome data for functional analysis of the predicted nat-siRNAs, we further investigated the targets reported by the root nat-siRNAs. We found that out of the 63 reported targets, 31, 12 and 1 were also found in seedling, leaf and flower, respectively, suggesting that nat-siRNAs may play both tissue-specific and wide-spread roles.

## DISCUSSION

Small RNAs that originate from endogenous RNA transcripts that share sequence complementary to other RNA transcript sequences are termed nat-siRNAs, and like miRNAs, they have been shown to regulate the translation of specific mRNAs through mRNA cleavage (5). Recently, there has been increase in the amount of research focused on classifying this type of sRNA and investigating their possible function. Even so, bioinformatics tools designed to identify nat-siRNAs from high-throughput sequencing data are limited in both number and function. In this paper, we describe a new software tool and pipeline, called NATpare, which is able to perform analyses on recent sRNA sequencing datasets within a reasonable timeframe for the very first time. When compared against the only available tool for this type of analysis, NATpare achieved a speed-up of 227× (1 day, 18 h and 34 min compared to just 11 min and 15 s) when benchmarked on a simulated *A. thaliana* dataset. In addition, NATpare was able to complete all analyses of the simulated non-model organism datasets, including *T. aestivum* which took just 13 h and 2 min, whereas NATpipe was unable to complete any non-model organism analysis within the 10 day cut-off. Prediction performance benchmarking of NATpare demonstrated its ability capture a larger number of previously reported nat-siRNAs in *G. max* when compared with NATpipe and further investigation into these results led us to identify that part of NATpipe's algorithm was causing some known *cis*-NAT pairs to be discarded.

In this study, we exemplified the usage of NATpare by performing analyses on data obtained from plants as the primary mechanism for RNA silencing in plants is mRNA cleavage, whereas in animals the primary mechanism is translational repression. However, degradome data has also been obtained in animal systems, for example in human (43) and mouse (44), and so, in principle, NATpare could also be used to analyse animal data.

The founding examples of nat-siRNAs were in *A. thaliana* seedling, where a pair of *cis*-NATs, SRO5 and P5CDH, were shown to be involved in salt tolerance through the RNAi pathway (5). We demonstrated the use of NATpare by performing analysis on a publicly available *A. thaliana* seedling dataset (33), consisting of control and salt stress libraries, followed by a DE analysis on the reported nat-siRNAs. Intriguingly, NATpare did not capture the same salt stress responsive nat-siRNAs as reported in a previous study (5) and further investigation showed that the previously found sequences were not present within the more recent salt stress dataset that we analysed. However, we did identify a number of upregulated nat-siRNAs in salt stress treated *A. thaliana* seedling whose originating transcripts are currently annotated as either potential natural antisense genes, unknown protein or simply described as other RNA. These results suggest that more work is required into the role of these sRNAs in salt stress and also additional work into whether nat-siRNAs are specific to salt stress or indeed play a responsive role in plants under various stress conditions. However, based on previous findings (5), the function of these upregulated nat-siRNAs may be to ensure the downregulation of the corresponding protein coding transcripts contained within the NAT pair. Additionally, the identification of nat-siRNAs originating from transcripts where the annotation is currently unknown, for example AT3G41762.1, may enable additional annotation information to be included, similar to AT1G05562.1, which is labelled as a potential natural antisense gene in the current annotation.

In plants, post-transcriptional regulation by sRNAs usually result in mRNA cleavage and subsequent degradation. Degradome data is a useful resource for identifying the potential function of a sRNA as it captures the uncapped 5’ ends of cleaved mRNAs for sequencing, which can then be aligned back to the reference transcripts and used to identify causal sRNA(s). We used a combination of NATpare and PAREsnip2 on the *A. thaliana* D3 dataset to predict and identify the possible targets of nat-siRNAs that were conserved between two biological replicates in flower, leaf, root and seedling tissues. In this analysis, we identified a number of interactions, conserved between replicates, which were found to be either tissue-specific or present within multiple of the analysed tissues. However, as these results are based solely on predictions, further experimental validation is necessary to determine the exact role or function that these nat-siRNAs play. Nonetheless, bioinformatics approaches to identify possible targets from sequencing data and subsequent validation is a vital step in understanding the function of a sRNA. Thus, we hope that the development of NATpare will lead to further understanding of the origin and function of nat-siRNAs in all manner of experimental contexts.

## DATA AVAILABILITY

NATpare is available as part of the UEA sRNA Workbench (25) and can be downloaded from http://srna-workbench.cmp.uea.ac.uk/. Additionally, the source code has been released on GitHub and is accessible at https://github.com/sRNAworkbenchuea/UEA_sRNA_Workbench/.

## Supplementary Material

gkaa448_Supplemental_Files
