# Supplementary figures and images for: NATpare: a pipeline for high-throughput prediction and functional analysis of nat-siRNAs
Source: Nucleic Acids Res. 2020 May 28;48(12):6481–90. doi: 10.1093/nar/gkaa448 (PMC7337908; doi:10.1093/nar/gkaa448)

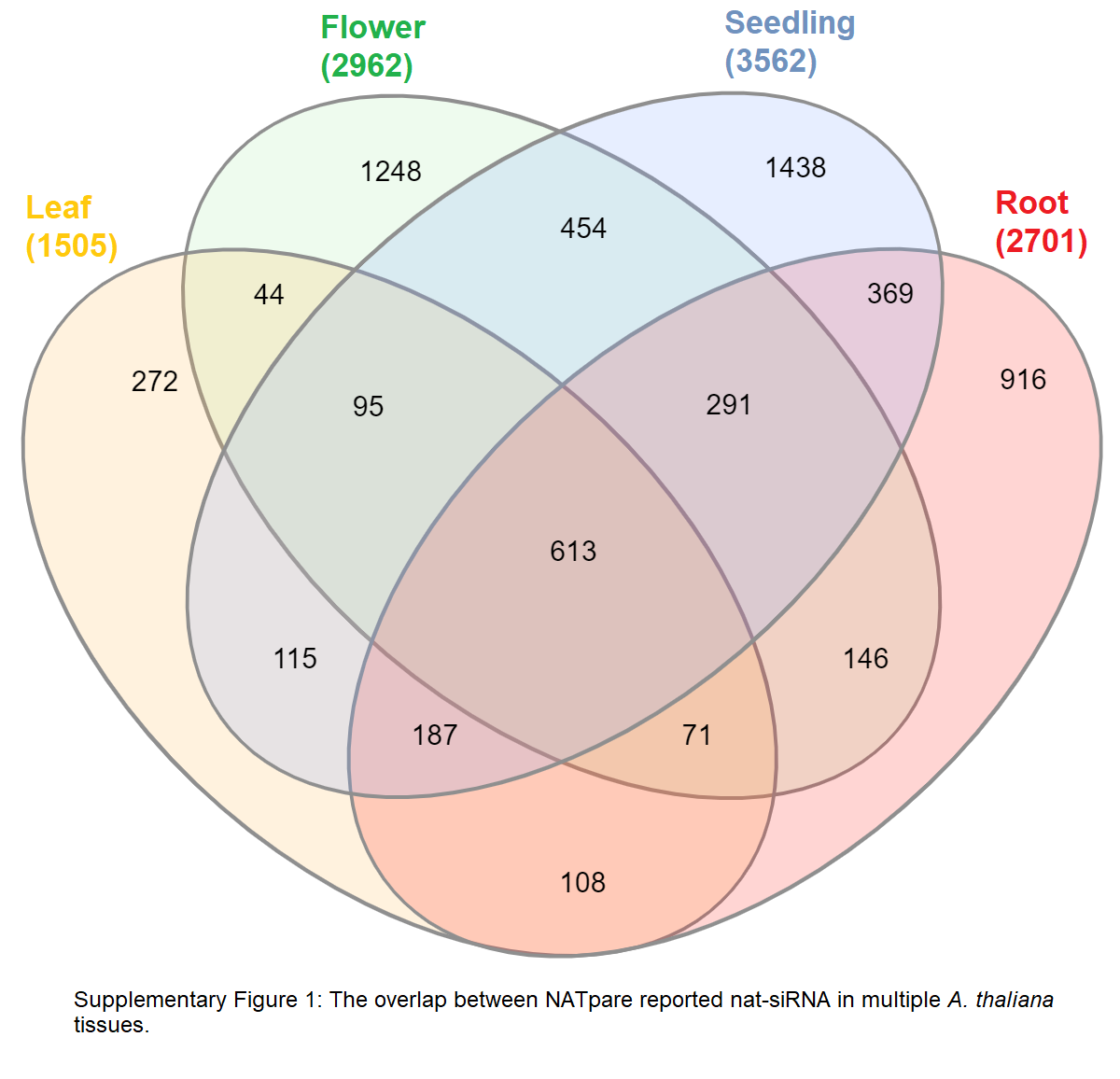

Supplement: gkaa448_Supplemental_Files [file gkaa448_supplemental_files.zip › SupplementaryFigure1.png]

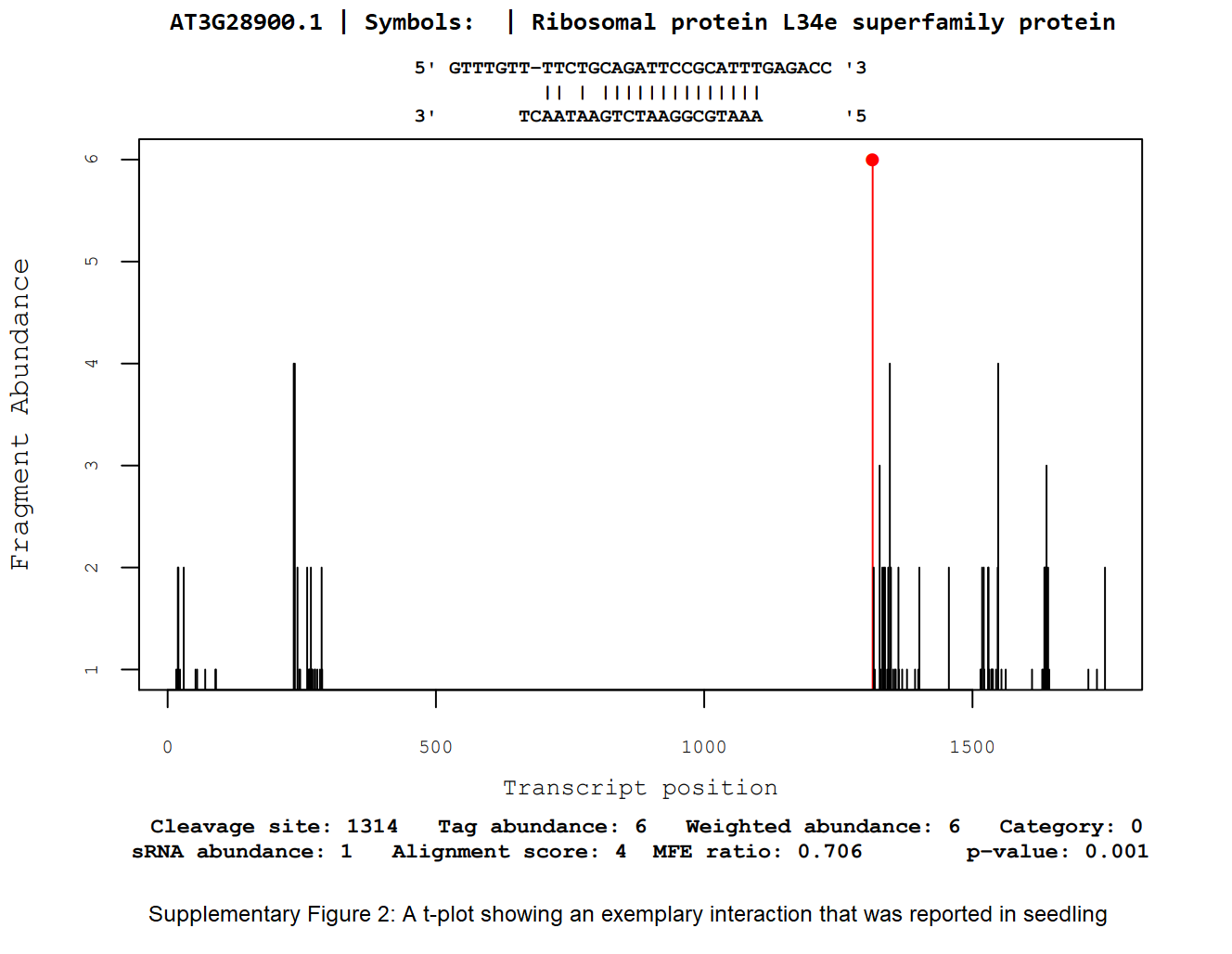

Supplement: gkaa448_Supplemental_Files [file gkaa448_supplemental_files.zip › SupplementaryFigure2.png]
